# The Prevalence and Symptom Profile of Premenstrual Syndrome Among Medical Students in a South Indian Medical College: A Cross-Sectional Study

**DOI:** 10.7759/cureus.98972

**Published:** 2025-12-11

**Authors:** Aiswaryalakshmi S Raja, Akila Govindarajan Venguidesvarane, Kiruthiga. T Thangasamy

**Affiliations:** 1 Community Medicine, Sri Ramachandra Institute of Higher Education and Research, Chennai, IND; 2 Obstetrics and Gynecology, Sri Ramachandra Institute of Higher Education and Research, Chennai, IND

**Keywords:** cross sectional study, medical students, premenstrual syndrome, prevalence, public health

## Abstract

Introduction

Premenstrual syndrome (PMS) is a prevalent yet underrecognized gynecological condition among women of reproductive age. It is characterized by physical, emotional, and behavioral changes that occur in the luteal phase of the menstrual cycle. Among medical students, who often experience substantial academic and emotional stress, these symptoms can significantly affect their well-being, academic performance, and daily functioning.

Objective

The primary objective was to estimate the prevalence of premenstrual syndrome (PMS) among female medical students. The secondary objectives were to assess the severity of PMS symptoms and to examine the timing of affective and somatic symptoms in relation to the menstrual cycle.

Methodology

This cross-sectional study was conducted among 190 medical students in a South Indian medical college. Data were gathered through face-to-face interviews using the premenstrual syndrome questionnaire, which covers demographic details and premenstrual syndrome symptoms. Analysis was done using IBM Corp. Released 2014. IBM SPSS Statistics for Windows, Version 20. Armonk, NY: IBM Corp. Descriptive statistics were represented in numbers and percentages.

Results

The overall prevalence of PMS was 135 (71.1%). Affective symptoms such as irritability was 181 (95.3%), mood swings was 176 (92.6%), sadness was 155 (81.6%), and anxiety was 123 (64.7%) were highly prevalent, while somatic symptoms including fatigue 172 was (90.5%), backache was 156 (82.1%), acne was 146 (76.8%), abdominal bloating was 140 (73.7%), and headache was 113 (59.5%) were also frequently reported. Among those experiencing symptoms, the majority, 124 (65.3%), had moderate severity, while 32 (16.8%) had severe symptoms and 34 (17.9%) had mild symptoms. Severe symptoms were most pronounced for abdominal cramps, 83 (43.7%); mood swings, 62 (32.6%); and irritability, 60 (31.6%). Most symptoms were present during the premenstrual phase, with only a few persisting beyond menstruation.

Conclusion

PMS is widespread among young adult women in academic settings, with a substantial burden of emotional and somatic symptoms. These symptoms can impact both personal well-being and academic performance. Improving awareness, incorporating menstrual health education, and ensuring access to timely medical and psychological support could help students cope better and improve both health and academic outcomes.

## Introduction

Premenstrual syndrome (PMS) refers to a range of psychological and somatic symptoms occurring during the luteal phase of the menstrual cycle [1]. These symptoms often result in considerable distress and impairment of daily functioning [2]. PMS is marked by emotional, behavioral, and physical symptoms that can severely disrupt interpersonal relationships and the overall quality of life [3,4].

Symptoms of PMS include irritability, mood swings, anger, crying spells, breast tenderness, nausea, appetite changes, weight gain, abdominal cramps, anxiety, back pain, headaches, constipation, sadness, fatigue, and swelling of the lower extremities [5]. Marked variability exists in the frequency and severity of PMS symptoms, and multiple population-based studies have evaluated PMS by examining the pattern and intensity of symptoms experienced during the premenstrual phase [6].

Globally, up to 75% of women of reproductive age experience PMS [7]. In India, the prevalence varies significantly: it was found to be 86% among college-going students [8], with pooled prevalence estimates for PMS and premenstrual dysphoric disorder (PMDD) being 43% and 8%, respectively [9]. Among adolescents, the prevalence has been reported as 49.6% [4], while a study conducted in Puducherry documented a prevalence of 62.7% [10].

The exact etiology of PMS remains unclear. Hormonal fluctuations, particularly estrogen dominance and progesterone deficiency, are often implicated. Moreover, altered serotonergic activity has been recognized as a key contributor [11]. Estrogen withdrawal may increase hypothalamic norepinephrine release, leading to a subsequent reduction in serotonin, dopamine, and acetylcholine levels, which leads to fatigue, depression, and insomnia [12,13]. Recent research also highlights the potential role of neurosteroids like allopregnanolone in the pathophysiology of PMS [14].

Despite its widespread occurrence, awareness of PMS remains low, particularly in South India. Many women fail to correlate their symptoms with the menstrual cycle. Hence, this study aims to estimate the prevalence of PMS and to examine the severity and timing of associated symptoms among medical students at a tertiary care center in South India.

## Materials and methods

Study design

This research was conducted as a cross-sectional study aimed at determining the prevalence and symptom profile of premenstrual syndrome (PMS) among female medical students.

Study setting

The study was carried out at a medical college in South India, involving undergraduate students enrolled in the third and fourth years of the MBBS program. 

Study duration

The study was conducted over a three-month period, from August 2024 to October 2024, coinciding with the regular academic schedule to ensure adequate student participation.

Sample size and sampling method

The prevalence of premenstrual syndrome reported by Upadhyay et al. [3] was used for calculating the sample size, which was determined to be 186 using the formula

n =$\frac{Z^2pq}{d^2}$

where prevalence (p) = 86%, absolute precision (d) = 5%, and confidence interval = 95%.

Sampling population

Inclusion and Exclusion Criteria

Inclusion: third- and fourth-year female medical students in a South Indian medical college who provided informed consent.

Exclusion: students on psychiatric medication, those with psychiatric illnesses, diabetes, or hypertension, or those unwilling to participate.

After obtaining permission from the concerned authorities, all third- and fourth-year female medical students were invited to participate. Only third- and fourth-year female medical students were included because they were the most accessible cohort during the study period, enabling uniform and feasible face-to-face data collection. Out of a total of 500 students, approximately 265 (53%) were female. Among them, 225 students responded (response rate: 85%). After applying the exclusion criteria, 190 participants were included in the final analysis. Students were excluded for the following reasons: use of psychiatric medication (n = 17), diagnosed psychiatric illness (n = 9), diabetes mellitus (n = 3), and hypertension (n = 6). Data collection was carried out through structured face-to-face interviews using a predesigned questionnaire.

Study tool

Data were collected using a Premenstrual Syndrome Questionnaire (Appendix), which was content reviewed by three obstetricians and gynecologists and pilot-tested among a small group of students for reliability and comprehension. The questionnaire assessed demographic characteristics and PMS-related symptoms. PMS was classified using a severity-based operational definition and was used to assess affective and somatic symptoms. Each item was rated as mild (1), moderate (2), severe (3), or not experienced (99). For the purpose of analysis, participants were considered to have PMS if they reported at least one affective symptom and at least one somatic symptom with a severity score of ≥2 (moderate or severe).

Demographic details

The first section of the questionnaire collected demographic and background variables, including age, contraceptive use, height, and weight. Body mass index (BMI) was later calculated from the recorded height and weight during data analysis. Participants who reported using contraceptives were asked to specify the type (e.g., oral pills, intrauterine device, or barrier method) and duration of use, as these factors can influence menstrual patterns and PMS symptoms.

Assessment of symptoms

The questionnaire consisted of six sections based on different symptoms. The symptoms included anxiety, irritability, mood swings, nervousness, sadness, crying, forgetfulness, insomnia, increased appetite, headache, fatigue, dizziness/fainting, palpitations, weight gain, swollen lower extremities, breast tenderness, abdominal bloating, oily skin, acne, constipation, diarrhea, backache, pain radiating down the thighs, abdominal cramps, and backache. Each symptom was rated (mild, moderate, or severe) and categorized by timing (before period, after period, or both).

Data analysis

Data was entered into Microsoft Excel (Redmond, USA) and analyzed using IBM Corp. Released 2014. IBM SPSS Statistics for Windows, Version 20. Armonk, NY: IBM Corp. software. Descriptive statistics were represented in numbers and percentages.

Ethical considerations

The study was approved by the Institutional Ethics Committee with Approval No. CSP-III/24/MAR/03/116. Written informed consent was obtained from all participants. All interviews were conducted individually in a private setting, no identifying information was collected, and all responses were anonymized to ensure participant privacy and confidentiality. Participants were informed that their responses would remain anonymous and confidential, which helped reduce the likelihood of response bias.

Reliability of the PMS questionnaire

The internal consistency of the PMS questionnaire was evaluated using Cronbach’s alpha to assess reliability within each symptom domain. The affective domain demonstrated good reliability (α = 0.84), while the somatic domain showed excellent reliability (α = 0.87). These values indicate strong internal consistency, confirming that the items within each domain reliably measure the intended constructs.

## Results

Demographic and anthropometric profile

A total of 190 female medical students participated in the study. The mean age was 21.4 ± 1.2 years. BMI assessment based on Indian criteria showed 5.79% underweight (n = 11), 35.79% (n = 68) normal weight, 13.16% (n = 25) overweight, and 45.26% (n = 86) obese (mean BMI = 25.11 kg/m²).

Regarding contraceptive use, only 6.8% (n = 13) of participants reported using some form of contraception, predominantly oral contraceptive pills, while the majority (93.2%, n = 177) were not using any contraceptive method at the time of the survey.

Prevalence and frequency of PMS symptoms

Using the predefined severity-based operational definition, among 190 participants, 135 (71.1%) met the criteria for PMS. These individuals reported at least one affective and one somatic symptom rated as moderate or severe. The remaining 55 (28.9%) did not meet the combined severity threshold and were classified as non-PMS.

**Figure 1:**
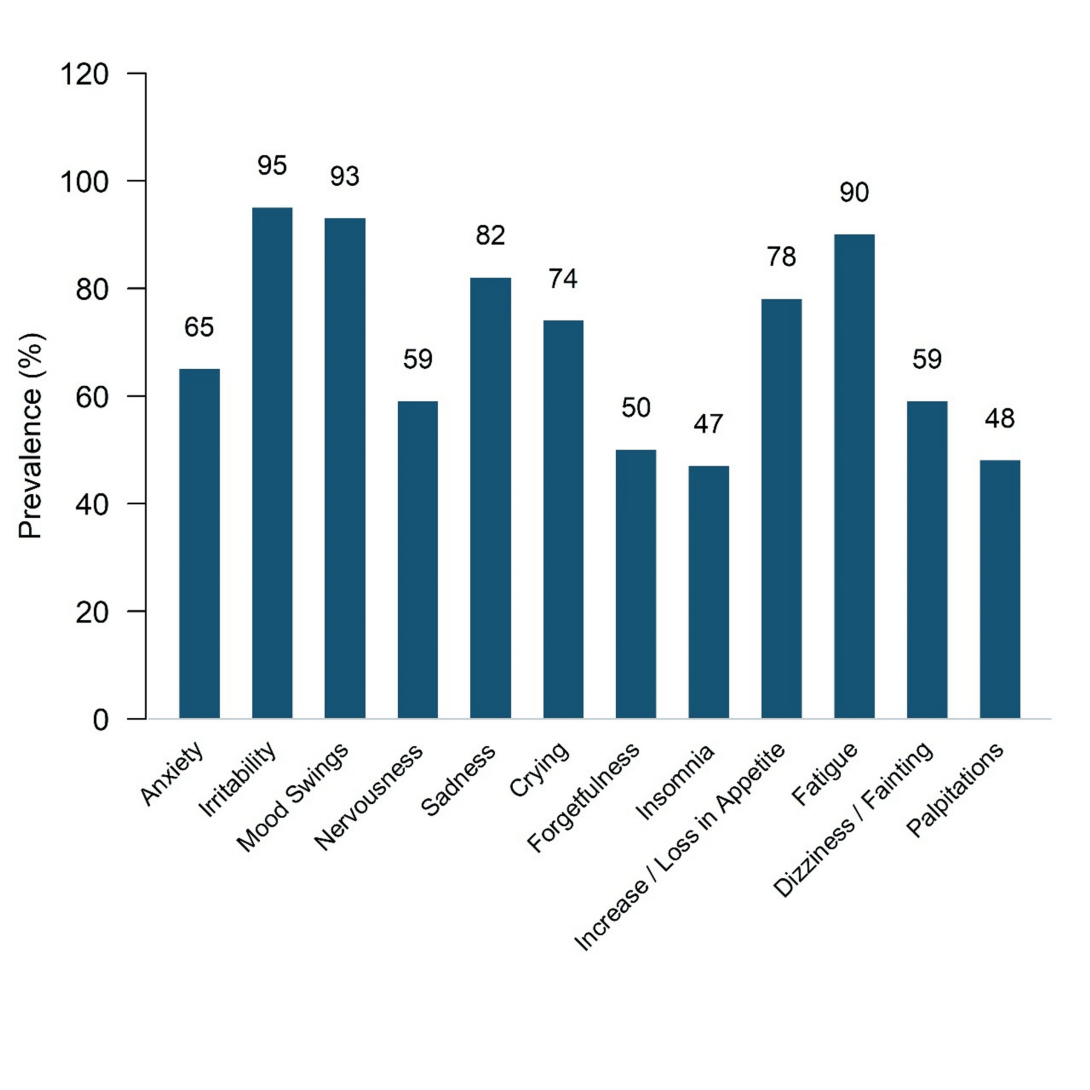
Distribution of Affective Symptoms Among Study Participants

The reported symptoms were divided into affective and somatic categories. Among affective symptoms (Figure 1), irritability (181 [95.3%]) and mood swings (176 [92.6%]) were the most frequently reported, followed by sadness (155 [81.6%]), crying spells (141 [74.2%]), and an increase in appetite (148 [77.9%]). Anxiety (123 [64.7%]), nervousness (112 [58.9%]), forgetfulness (96 [50.5%]), insomnia (90 [47.4%]), and palpitations (77 [40.5%]) were comparatively less common. These findings highlight the substantial emotional and behavioral disturbances associated with PMS in this group.

**Figure 2:**
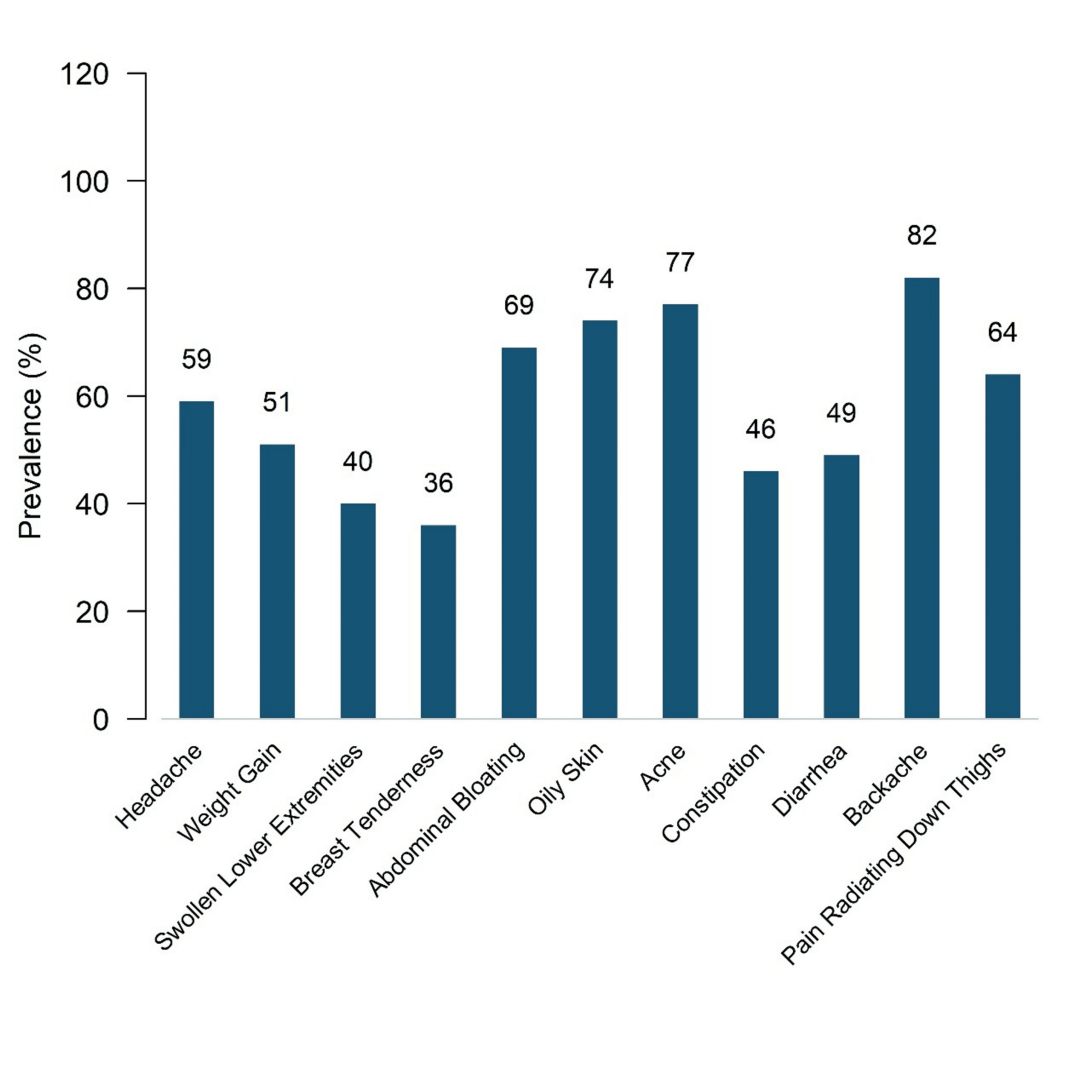
Distribution of Somatic Symptoms Among Study Participants

Among somatic symptoms (Figure 2), the most common complaints included abdominal cramps (175 [92.1%]), fatigue (172 [90.5%]), backache (156 [82.1%]), acne (146 [76.8%]), abdominal bloating (140 [73.7%]), and breast tenderness (132 [69.5%]). Headache (113 [59.5%]), pain radiating down the thighs (122 [64.2%]), and oily skin (112 [58.9%]) were also frequently reported. Weight gain (97 [51.1%]), diarrhea (93 [49.0%]), dizziness/fainting (92 [48.4%]), constipation (88 [46.3%]), and swelling of lower extremities (69 [36.3%]) were comparatively less prevalent.

Overall, both affective and somatic symptoms were highly prevalent, showing the complex impact of PMS on emotional, physical, and functional well-being among female medical students.

Severity of PMS symptoms

The severity of premenstrual symptoms among the participants is presented in Tables 1, 2. Among affective symptoms, irritability (108 [56.8%] mild, 60 [31.6%] severe), mood swings (65 [34.2%] mild, 62 [32.6%] severe), and an increase in appetite (46 [24.2%] mild, 57 [30.0%] severe) showed the highest severe symptom rates. Sadness (52 [27.5%] mild, 53 [27.9%] severe) and crying (51 [26.8%] mild, 55 [28.9%] severe) were also significantly reported. Other affective complaints, such as anxiety (56 [29.5%] mild, 22 [11.6%] severe), nervousness (60 [31.6%] mild, 23 [12.1%] severe), forgetfulness (59 [31.1%] mild, 13 [6.8%] severe), insomnia (50 [26.3%] mild, 21 [11.1%] severe), and palpitations (49 [25.8%] mild, 10 [5.3%] severe), were predominantly experienced at mild to moderate levels.

**Table 1:** Severity of Affective Premenstrual Symptoms Among College-Going Girls (n = 190)

Symptom	Mild (n, %)	Moderate (n, %)	Severe (n, %)	Not Experienced (n, %)
Anxiety	56 (29.5)	45 (23.7)	22 (11.6)	67 (35.3)
Irritability	108 (56.8)	13 (6.8)	60 (31.6)	9 (4.7)
Mood Swings	65 (34.2)	49 (25.8)	62 (32.6)	14 (7.4)
Nervousness	60 (31.6)	29 (15.3)	23 (12.1)	78 (41.1)
Sadness	52 (27.5)	50 (26.3)	53 (27.9)	35 (18.4)
Crying	51 (26.8)	35 (18.4)	55 (28.9)	49 (25.8)
Forgetfulness	59 (31.1)	24 (12.6)	13 (6.8)	94 (49.5)
Insomnia	50 (26.3)	19 (10.0)	21 (11.1)	100 (52.6)
Increase in Appetite	46 (24.2)	45 (23.7)	57 (30.0)	42 (22.1)
Palpitations	49 (25.8)	18 (9.5)	10 (5.3)	113 (59.5)

**Table 2:** Severity of Somatic Premenstrual Symptoms Among College-Going Girls (n = 190)

Symptom	Mild (n, %)	Moderate (n, %)	Severe (n, %)	Not Experienced (n, %)
Headache	73 (38.4)	15 (7.9)	25 (13.2)	77 (40.5)
Fatigue	101 (53.2)	26 (13.7)	45 (23.7)	18 (9.5)
Dizziness / Fainting	57 (30.0)	22 (11.6)	13 (6.8)	98 (51.6)
Weight gain	57 (30.0)	26 (13.7)	14 (7.4)	93 (49.0)
Swollen lower extremities	57 (30.0)	7 (3.7)	5 (2.6)	121 (63.7)
Breast tenderness	69 (36.3)	33 (17.4)	30 (15.8)	58 (30.5)
Abdominal bloating	53 (27.9)	40 (21.1)	47 (24.7)	50 (26.3)
Oily skin	54 (28.4)	30 (15.8)	28 (14.7)	78 (41.1)
Acne	61 (32.1)	50 (26.3)	35 (18.4)	44 (23.2)
Constipation	59 (31.1)	19 (10.0)	10 (5.3)	102 (53.6)
Diarrhea	55 (29.0)	25 (13.2)	13 (6.8)	97 (51.1)
Backache	67 (35.3)	44 (23.2)	45 (23.7)	34 (17.9)
Pain radiating down thighs	44 (23.2)	40 (21.1)	38 (20.0)	68 (35.8)
Abdominal Cramps	30 (15.8%)	62 (32.6%)	83 (43.7%)	15 (7.9%)

Regarding somatic symptoms, abdominal cramps (30 [15.8%] mild, 83 [43.7%] severe) showed the most pronounced severity, followed by fatigue (101 [53.2%] mild, 45 [23.7%] severe), abdominal bloating (53 [27.9%] mild, 47 [24.7%] severe), backache (67 [35.3%] mild, 45 [23.7%] severe), and headache (73 [38.4%] mild, 25 [13.2%] severe). Additional physical complaints included breast tenderness (69 [36.3%] mild, 30 [15.8%] severe); acne (61 [32.1%] mild, 35 [18.4%] severe); weight gain (57 [30.0%] mild, 14 [7.4%] severe); dizziness/fainting (57 [30.0%] mild, 13 [6.8%] severe; and pain radiating down the thighs (44 [23.2%] mild, 38 [20.0%] severe), oily skin (54 [28.4%] mild, 28 [14.7%] severe), constipation (59 [31.1%] mild, 10 [5.3%] severe), diarrhea (55 [29.0%] mild, 13 [6.8%] severe), and swollen lower extremities (57 [30.0%] mild, 5 (2.6%) severe).

As shown in Table 3, among the 190 participants, 34 (17.9%) reported mild, 124 (65.3%) reported moderate, and 32 (16.8%) experienced severe overall PMS symptoms. Overall, our findings suggest that abdominal cramps emerged as the symptom with the highest severe intensity, 83 (43.7%), followed by mood swings, 62 (32.6%), and irritability, 60 (31.6%). This indicates that PMS has a significant impact on both the physical and emotional well-being of women.

**Table 3:** Severity Frequency of Symptoms Among College-Going Girls (n = 190)

Severity Category	Frequency (n)	Percentage (%)
Mild	34	17.9
Moderate	124	65.3
Severe	32	16.8
Total	190	100.0

Timing of premenstrual symptoms relative to menstrual onset

Table 4 demonstrates that the majority of affective symptoms predominantly occur before the onset of menstruation. Anxiety, nervousness, sadness, and insomnia were reported by over 85% of participants prior to periods. Mood swings, crying, and irritability showed persistence across both phases, indicating that these emotional changes are not limited to the premenstrual phase but could also occur after menstruation. Overall, affective symptoms are highly present in the premenstrual phase and have persistence afterwards. 

**Table 4:** Timing of Affective PMS Symptoms Relative to Menstrual Onset PMS: Premenstrual syndrome

Symptom	Before period n (%)	After period n (%)	Before & after period n (%)	Total n
Anxiety	114 (92.7)	–	9 (7.3)	123
Irritability	132 (72.9)	45 (24.9)	4 (2.2)	181
Mood Swings	146 (83.4)	7 (4.0)	22 (12.6)	175
Nervousness	105 (93.8)	2 (1.8)	5 (4.5)	112
Sadness	139 (89.7)	3 (1.9)	13 (8.4)	155
Crying	125 (88.7)	3 (2.1)	13 (9.2)	141
Forgetfulness	83 (86.5)	8 (8.3)	5 (5.2)	96
Insomnia	81 (90.0)	4 (4.4)	5 (5.6)	90
Increase in Appetite	123 (83.1)	11 (7.4)	14 (9.5)	148
Palpitations	69 (89.6)	4 (5.2)	4 (5.2)	77

Table 5 illustrates that most somatic symptoms are highly prevalent before the period, with breast tenderness (125 [94.7%]), acne (134 [91.8%]), oily skin (103 [92.0%]), and swollen lower extremities (69 [100%]) reported most frequently. Headache, fatigue, and dizziness were occasionally observed after the period, but overall, the physical complaints are more concentrated in the premenstrual phase. Certain somatic symptoms, such as abdominal bloating, backache, and pain radiating down the thighs, persisted across both phases, indicating that while somatic symptoms are primarily premenstrual, some may extend into the menstrual or postmenstrual phase.

**Table 5:** Timing of Somatic PMS Symptoms Relative to Menstrual Onset PMS: Premenstrual syndrome

Symptom	Before period n (%)	After period n (%)	Before & after period n (%)	Total n
Headache	82 (72.6)	28 (24.8)	3 (2.6)	113
Fatigue	133 (77.3)	35 (20.4)	4 (2.3)	172
Dizziness / Fainting	83 (90.2)	6 (6.5)	3 (3.3)	92
Weight Gain	86 (88.7)	7 (7.2)	4 (4.1)	97
Swollen Lower Extremities	69 (100.0)	–	–	69
Breast Tenderness	125 (94.7)	3 (2.3)	4 (3.0)	132
Abdominal Bloating	124 (88.6)	5 (3.6)	11 (7.9)	140
Oily Skin	103 (92.0)	2 (1.8)	7 (6.3)	112
Acne	134 (91.8)	2 (1.4)	10 (6.8)	146
Constipation	76 (87.4)	7 (8.1)	4 (4.6)	87
Diarrhea	85 (91.4)	5 (5.4)	3 (3.2)	93
Backache	134 (85.9)	10 (6.4)	12 (7.7)	156
Pain Radiating Down Thighs	105 (86.1)	7 (5.7)	10 (8.2)	122

## Discussion

Our study provides insight into the prevalence and symptomatology of premenstrual syndrome (PMS) among final-year medical students in South India. High frequencies of individual symptoms such as irritability and mood swings were noted, although PMS based on severity was present in 71.1% of students. PMS has a substantial impact on women and should be addressed. The overall prevalence of PMS in this study (71.1%) is comparable to that reported among college-going women in other studies, such as the 86% observed by Upadhyay et al. [8] and the 62.7% reported by Bhuvaneswari et al. in Puducherry [10]. On a global scale, Direkvand-Moghadam et al. [7] reported a pooled prevalence of approximately 47.8%, indicating that the burden of PMS is consistently high worldwide, though somewhat higher in the Indian population. Our study is more crucial, as medical students experience lifestyle and psychosocial factors such as academic stress, irregular sleep, and erratic dietary patterns, which can influence hormonal balance and increase symptomatology.

According to the various studies done in South India, which include the Durairaj et al. study as well as the Karpagavalli et al. study [15,16], the following symptoms were the most prevalent: affective symptoms like irritability (96%) and somatic symptoms like body aches (71.3%) and abdominal heaviness (64.3%) with fatigue/lack of energy (94.7%). Additionally, a study in Thanjavur noted that swollen extremities, palpitations, insomnia, and crying were the least prevalent symptoms [17]. A study in Himachal Pradesh also noted insomnia to be the least prevalent symptom. All these findings are consistent with our findings [18].

The high prevalence of affective symptoms such as irritability, sadness, and crying highlights the neuropsychological basis of PMS. Fluctuations in estrogen and progesterone levels during the luteal phase affect neurotransmitter pathways, particularly serotonin and gamma-aminobutyric acid (GABA), which play crucial roles in emotional regulation [12,19-20]. Reduced serotonergic activity may explain the predominance of psychological symptoms such as anxiety, depression, and irritability. Moreover, emerging evidence suggests a role for neurosteroids like allopregnanolone, a metabolite of progesterone that modulates GABA-A receptors and can influence mood, irritability, and cognitive function [14].

On the other hand, the Thanjavur study quoted that 97% of the participants experienced breast tenderness. Moreover, somatic symptoms like abdominal bloating, weight gain, and fluid retention were also found to be above average, which was attributed to regional dietary habits, hydration levels, or environmental differences such as climate [17]. These symptoms do not corroborate our findings. According to the Chauhan et al. study, in a rural Uttar Pradesh population, somatic symptoms like headaches (66%), acne (60%), and generalized body aches (57%) were the predominant symptoms. Our study did not reflect similar prominence for headaches or acne, due to differences in water quality, nutrition, or physical exertion between rural and urban academic settings [21].

Postmenstrual symptoms were markedly fewer. Headaches (28 [24.78%]), irritability (45 [24.86%]), and fatigue (35 [20.35%]) were the only symptoms with notable persistence after menstruation, suggesting delayed hormonal stabilization in some participants. This aligns with the findings of the Thanjavur study, where fluid retention and fatigue extended beyond menstruation in a minority of students [17]. These residual symptoms may reflect either hormonal sensitivity or stress-related carryover effects, which may be due to the medical curriculum, an added stressor that could be influencing the hormonal activity.

A small subset of participants reported symptoms persisting both before and after menstruation. Affective symptoms like mood swings (22 [12.57%]), crying spells (13 [9.15%]), and sadness (13 [8.39%]) were the most commonly persistent symptoms across both phases. As mentioned in Modzelewski et al.’s review of PMS etiology, the role of GABAergic neurosteroids like allopregnanolone in mood modulation may be responsible for these symptoms. It is also noted that lifestyle factors like poor sleep, caffeine intake, and stress may increase severity, causing these differences [14].

This study highlights the importance of premenstrual syndrome and its significant impact on women. It is important to understand what a woman goes through in these trying times and how to tackle each symptom. Most women are unaware of what symptoms they are experiencing and why they go through them. Our study supersedes other studies by analyzing both the severity and phase-wise occurrence of symptoms, which provides more well-supported data. This offers more practical clinical value. Understanding when symptoms occur helps doctors design timing-specific interventions, such as starting pharmacotherapy or stress-reduction techniques during the luteal phase. For medical students, this information can guide academic and personal planning, potentially reducing exam-related performance lag caused by PMS. By highlighting that most symptoms concentrate in the premenstrual phase and resolve shortly after menstruation begins, the study helps distinguish PMS from other menstrual or psychiatric disorders. In addition, the study was conducted on a one-to-one basis, which made it easier to go deeper into each symptom and collect more information.

While our study provides valuable insights, it has its own limitations. As the study was conducted at a single medical college using a non-random, convenience sampling approach, the findings may have limited generalizability to wider populations. Rare cases may be hard to find with a limited sample size. In addition, symptom reporting was based on participant recall, which may lead to recall bias. Face-to-face interviews, although structured, may have encouraged socially desirable responses, which may have led to social desirability bias.

## Conclusions

PMS is highly prevalent among medical students, with significant physical and psychological symptoms, particularly mood-related complaints, with the most prevalent being irritability, mood swings, fatigue, and abdominal cramps. Most symptoms occur premenstrually, with some persisting beyond menstruation. These findings emphasize the urgent need for greater awareness, early recognition, and supportive interventions ranging from lifestyle measures to medical support in order to alleviate the impact of PMS on the quality of life and academic performance of medical students.

## Appendices

Premenstrual syndrome questionnaire

Patient Information

**Table 6:** Patient information

Field	Response
Full Patient Name	
Date	
Age	
Height	
Weight	
Present Contraception	None/Pill/IUD/DMPA/Other
History of Contraceptive Pills	Yes/No
Number of Years	

Symptom Assessment

Instructions: Rate symptoms according to severity (1 = Mild, 2 = Moderate, 3 = Severe) and indicate timing (Week Before Period/Week After Period/Others/Nil)

**Table 7:** Symptom assessment table

Category	Symptom	Severity (1-3)			Week Before Period	Week After Period	Others/Nil
Affective Symptoms	Anxiety	1	2	3			
	Irritability	1	2	3			
	Mood Swings	1	2	3			
	Nervousness	1	2	3			
	Sadness	1	2	3			
	Crying	1	2	3			
	Forgetfulness	1	2	3			
	Insomnia	1	2	3			
	Increase/Loss in appetite	1	2	3			
	Fatigue	1	2	3			
	Dizziness/Fainting	1	2	3			
	Palpitations	1	2	3			
Somatic Symptoms	Headache	1	2	3			
	Weight Gain	1	2	3			
	Swollen Lower Extremities	1	2	3			
	Breast Tenderness	1	2	3			
	Abdominal Bloating	1	2	3			
	Oily Skin	1	2	3			
	Acne	1	2	3			
	Constipation	1	2	3			
	Diarrhea	1	2	3			
	Backache	1	2	3			
	Pain Radiating Down Thighs	1	2	3			
